# The association of selected multiple sclerosis symptoms with disability and quality of life: a large Danish self-report survey

**DOI:** 10.1186/s12883-021-02344-z

**Published:** 2021-08-16

**Authors:** S. Gustavsen, A. Olsson, H. B. Søndergaard, S. R. Andresen, P. S. Sørensen, F. Sellebjerg, A. Oturai

**Affiliations:** 1 Danish Multiple Sclerosis Center, Department of Neurology, Copenhagen University Hospital - Rigshospitalet, Glostrup, Denmark; 2 Department of Nuclear Medicine, Aalborg University Hospital, Aalborg, Denmark; 3 Department of Clinical Medicine, University of Copenhagen, Copenhagen, Denmark

**Keywords:** MS, QoL, Prevalence, Symptom burden

## Abstract

**Background:**

People with multiple sclerosis (MS) experience a wide range of unpredictable and variable symptoms. The symptomatology of MS has previously been reported in large sample registry studies; however, some symptoms may be underreported in registries based on clinician-reported outcomes and how the symptoms are associated with quality of life (QoL) are often not addressed.

The aim of this study was to comprehensively evaluate the frequency of selected MS related symptoms and their associations with disability and QoL in a large self-report study.

**Methods:**

We conducted a cross-sectional questionnaire survey among all patients at the Danish Multiple Sclerosis Center, Copenhagen University Hospital, Denmark. The questionnaire included information on clinical and sociodemographic characteristics, descriptors of QoL and disability, as well as prevalence and severity of the following MS symptoms: impaired ambulation, spasticity, chronic pain, fatigue, bowel and bladder dysfunction, and sleep disturbances.

**Results:**

Questionnaires were returned by 2244/3606 (62%). Participants without MS diagnosis or incomplete questionnaires were excluded, *n* = 235. A total of 2009 questionnaires were included for analysis (mean age 49.4 years; mean disease duration 11.7 years; and 69% were women).

The most frequently reported symptoms were bowel and bladder dysfunction (74%), fatigue (66%), sleep disturbances (59%), spasticity (51%) and impaired ambulation (38%). With exception of fatigue and sleep disturbances, all other symptoms increased in severity with higher disability level. Invisible symptoms (also referred to as hidden symptoms) such as fatigue, pain and sleep disturbances had the strongest associations with the overall QoL.

**Conclusion:**

We found invisible symptoms highly prevalent, even at mild disability levels. Fatigue, pain and sleep disturbances had the strongest associations with the overall QoL and were more frequently reported in our study compared with previous registry-based studies. These symptoms may be underreported in registries based on clinician reported outcomes, which emphasizes the importance of including standardized patient reported outcomes in nationwide registries to better understand the impact of the symptom burden in MS.

## Background

Multiple sclerosis (MS) is an immune mediated neurological disease with malfunction of the immune system causing destruction of myelin sheaths and neuroaxonal damage in the central nervous system. The prevalence of people with MS (PwMS) in Denmark is 284 per 100,000 inhabitants and is among one of the highest in the world [1]. In addition, MS is the most frequent neurological disease leading to prolonged and progressive physical, psychological and cognitive disability in young adults. However, during the last decades the natural history of MS appears milder which probably is the result of an interplay between several factors including changes in the diagnostic criteria and MS neuroepidemiology, early and appropriate disease-modifying treatment and improvement of the general state of health [2].

MS is characterized by a wide variety of symptoms, including muscle spasticity and weakness, gait disturbances, chronic pain, fatigue, sleep disturbances, and bowel and bladder dysfunction. Previous studies have shown inconsistencies in the prevalence of MS-related symptoms and their impact on quality of life (QoL). A German registry study, which aimed to assess the prevalence of selected MS related symptoms, found that the most frequently reported symptoms, over the course of the disease, were fatigue (58%), spasticity (48%), voiding disorder (44%), ataxia/tremor (36%) and pain (34%) [3]. . Symptom prevalence tables established by Kister et al., based on data from the North American Research Committee on Multiple Sclerosis (NARCOMS), have shown that mobility (gait) impairment and severity of pain, spasticity, fatigue, and bowel and bladder dysfunction increased in frequency in association with increasing disease duration. Furthermore, mobility impairment and bowel and bladder dysfunction were reported by half of the patients within the first year of diagnosis [4]. With a prevalence of moderate or severe sleep problems of 51%, PwMS have significantly more sleep difficulties compared with the general population [5]. Sleep disturbances may be due to MS related symptoms such as pain, spasticity and voiding dysfunction, and furthermore be a contributing factor to fatigue [6]. This illustrates the wide-ranging symptomatology of MS, which consists of a complex interplay between physical, cognitive and mental symptoms.

QoL is significantly impaired in PwMS and closely associated with depression, fatigue and physical activity [7]. Zhang et al. found that fatigue, balance problems, sensory problems and walking difficulties were reported with the highest impact on QoL [8]. The data were obtained from an Australian longitudinal study database; however, the study included no information on symptom prevalence.

Most of the MS related symptoms have separately been associated with impaired QoL in previous studies, including pain [9], spasticity [10] and fatigue [11]. However, the studies have reported inconsistent results when examining the association between disability, primarily addressed by the Expanded Disability Status Scale (EDSS) score, and QoL. Recent studies have found an inverse correlation between EDSS and QoL [8, 12]. Overall, previous studies show a significant association between MS related symptoms and perceived QoL; in addition, sociodemographic factors also play a significant role. A study, which addressed the effects of economic disadvantages in PwMS on their QoL found that income played a significant part in the prediction of QoL [13]. Likewise, occupation and family status are significant predictors [7]. Not surprisingly, the prediction of QoL is multifactorial which emphasizes the importance of including sociodemographic characteristics in the statistical analysis when assessing associations between MS symptoms and QoL.

The symptomatology in MS and its association with disability and QoL has never been comprehensively investigated. This study aims to evaluate, in a large Danish self-report study, the prevalence and severity of a broad range of MS related symptoms and its association with disability and QoL in MS.

## Methods

### Survey and questionnaire

A cross-sectional questionnaire survey was conducted among all patients at the Danish Multiple Sclerosis Center (DMSC), University of Copenhagen, Rigshospitalet, Copenhagen, Denmark. Data were collected from a web-based questionnaire and included information on sociodemographics, lifestyle factors, MS characteristics, MS related symptoms and cannabis use. The survey was conducted from April 2018 through June 2018 and distributed to 3606 PwMS. Clinical data (sex distribution, age, MS phenotype and level of disability) from the included PwMS cohort have been compared with data from the Danish Multiple Sclerosis Registry and were found representative with the DMSC population registered in the registry [14]. Noticeably, the patient population at the DMSC are more commonly treated with disease-modifying therapies, are younger and less disabled compared to the overall Danish MS population. The questionnaire and the study population have been fully described, including the description and characteristics of the sociodemographic measures, in a previously published paper that investigated the prevalence of cannabis use among PwMS (see section ‘Availability of the questionnaire’) [14]. This report primarily focuses on MS-related symptoms, disability and the QoL measures.

*Disability status* was determined by Patient Determined Disease Steps (PDDS) [15]. This is primarily based on ambulation and consists of 9 descriptors ranging from a score of 0 to 8, and can briefly be described as follows: 0) Normal: Mild symptoms, mostly sensory, but do not limit activity; 1) Mild disability: Noticeable symptoms but only small effect on lifestyle; 2) Moderate disability: No limitations in walking ability but significant problems that limit daily activities in other ways; 3) Gait disability: Symptoms interfere with activities, especially walking. Usually do not need a cane or other assistance to walk; 4) Early cane: Cane or a single crutch for walking all the time or part of the time; 5) Late cane: Cane or crutch for walking 25 ft; 6) Bilateral support: Two canes, crutches or a walker for walking 25 ft; 7) Wheelchair/Scooter: Not able to walk 25 ft, even with support. Wheelchair is the main mobility form; and 8) Bedridden: Unable to sit in a wheelchair for more than one hour. For further information on PDDS visit www.narcoms.org/pdds.

The severity of *impaired ambulation* was based on the PDDS levels ranging from 3 to 8 and the PDDS scores from 0 to 2 was categorized as normal ambulation (no impaired ambulation).

If participants reported *spasticity* (muscle stiffness or spasms) the severity in the past 7 days was rated by the numeric rating scale from 0 to 10 (NRS-11): 0 = no spasticity to 10 = worst possible spasticity, and furthermore classified by Penn Spasm Frequency Scale (Graded from 0 to 4: 0 = No spasm; 1 = Mild spasm induced by stimulation; 2 = Infrequent full spasms occurring less than once per hour; 3 = Spasms occurring more than once per hour; and 4 = spasms occurring more than 10 times per hour) [16]. In addition, current spasticity treatment was recorded.

*Bowel and bladder function* were characterized with inspiration from the functional system score in the Expanded Disability Status Scale [17], and were categorized as follows: 0) normal; 1) mild dysfunction (mild hesitancy, urgency and/or constipation); 2) moderate dysfunction (moderate urinary hesitancy/retention/urgency/incontinence and/or must wear pads or alter lifestyle because of bowel dysfunction); 3) severe dysfunction (frequent urinary incontinence or intermittent self-catheterization, needs enemata or manual measures to evacuate bowels); 4) lost bowel or bladder function; and 5) lost bowel and bladder function. The participants chose the category that represented their symptoms/function the most.

*Chronic pain* was defined as daily or continuously recurring pain for more than 3 months. Of those who reported chronic pain the mean pain intensity during the past seven days was determined by NRS-11: 0 = no pain to 10 = worst imaginable pain. The seven pain descriptors (burning, painful cold, electric shocks, tingling, pins and needles, numbness, and itching) in the Douleur Neuropathique 4 (DN4) questionnaire were used to distinguish between neuropathic and non-neuropathic pain, and were assessed as yes/no. The occurrence of at least 3 descriptors is suggestive of neuropathic pain [18]. Without clinical examination the seven-item DN4 has been considered as the most satisfactory tool to determine if pain is neuropathic [19]. Both musculoskeletal and visceral pain were included in the category non-neuropathic pain.

*Fatigue severity* was measured by the Fatigue Severity Scale (FSS), a 9-item scale. Each item was rated on a scale from 1 to 7, 1 = not troubled with fatigue to 7 = highly troubled with fatigue, with a total score ranging from 9 to 63. The higher the score, the greater the fatigue severity. The following cut-off values were used: < 36 = no to mild fatigue; 36–52 = moderate fatigue; and > 52 = severe fatigue [20, 21]. Furthermore, the FSS score was divided into two major components inspired by the conceptual frameworks presented by Goodwin et al. [21] The first component, ‘General and mental’, is the sum of the scores from item 1,3,5,7,8 and 9 (total score of 42). The second component, ‘Physical’, is the sum of the scores from item 2,4 and 6 (total score of 21). The ratio of the two components, calculated by the sum of the item scores included in the component and divided by the components total score, was used to evaluate whether general and mental fatigue vs. physical fatigue had the highest impact on the total fatigue score. A value above 1 indicates that general and mental fatigue component has higher impact compared with the physical component on the overall fatigue score, and vice versa.

Intensity of *sleep disturbances* the past seven days was reported by NRS-11: 0 = no disturbances, 10 = worst imaginable disturbances.

*QoL* was estimated using QoL-BDS (Quality of Life Basic Data Set) [22]. The instrument includes three QoL descriptors: overall health (life situation); physical health; and mental health. Participants were asked how satisfied they were with their overall well-being in the past four weeks, as well as their physical and mental health. Satisfaction was ranked by NRS-11: 0 = completely dissatisfied to 10 = completely satisfied.

### Symptom severity categorization

Currently, there is no consensus on how to define cut-off points for varying levels of symptom severity for the NRS-11 [23]. We categorized symptom intensity in no to mild (NRS 0–3), moderate (NRS 4–7) and severe (NRS 8–10). For chronic pain and spasticity, the intensity was categorized: mild (NRS 1–3), moderate (NRS 4–7) and severe (NRS 8–10), because the participants previously had answered whether they had chronic pain or spasticity (yes/no).

### Statistics

Baseline characteristics are presented as means and standard deviations (SD) or medians and interquartile range (IQR, 25–75%) depending on normality. Normality was assessed by histograms, Q-Q plots and Shapiro-Wilks test. The numeric rating scale (NRS-11) was defined as a Likert-type scale and therefore primarily used in statistics models as an ordinal scale. However, some variables on the NRS-11 scale followed a normal distribution, why these variables were presented with means and SD. Correlation between variables was assessed by Spearman’s rank correlation coefficient (rho). The intensity measure of pain and spasticity was used in both the correlation analyses and the linear regression model.

To estimate the relative association of selected variables with the descriptors of QoL, a multivariable linear regression model was used. In this model the null hypothesis states that there is no relationship or effect between QoL descriptors and the variables. Variables were included in the model if previous studies had shown consistent, statistically significant associations. The regression model was refined by ensuring that the data were homoscedastic, had normally distributed residuals, had no major outliers, and were absent of multicollinearity. The variables were included in the model as characterized in Table 1. The model was validated and tested for overfitting by cross validation, which was possible due to the large sample size. The results were presented in standardize coefficient values (*beta*) due to varying variable scales. All statistical analyses were performed with IBM SPSS® (Statistical Product and Service Solutions) version 25**.**

**Table 1:** Clinical and sociodemographic characteristics of the study population

	Total, *n = 2009*
Age, years, mean (SD)	49.4 (12.1)
Sex, female, n (%)	1389 (69.1)
Diagnosis, n (%)	
Multiple sclerosis	1979 (98.5)
CIS (clinical isolated syndrome)	30 (1.5)
Clinical type of MS, n (%)	
Relapse-remitting MS (RRMS)	1383 (69.9)
Secondary progressive MS (SPMS)	153 (7.7)
Primary progressive MS (PPMS)	153 (7.7)
Unaware	290 (14.7)
Age at diagnosis, mean (SD)	37.7 (10.9)
Duration of diagnosis, years, mean (SD)	11.7 (8.1)
Current DMT, n (%)	1502 (75)
Duration of DMT use, years, median (ICR)	9 (4–14)
Quality of life, NRS-11^1^, mean (SD)	
Satisfaction with life situation	6.5 (2.5)
Satisfaction with physical health	5.6 (2.8)
Satisfaction with mental health	6.5 (2.6)
Sleep disturbances, NRS-11^2^, mean (SD)	4.2 (2.8)
FSS score, median (range)	43 (54)
No to mild fatigue (FSS score < 36), n (%)	728 (36)
Moderate fatigue (FSS score 36–52), n (%)	679 (34)
Severe fatigue (FSS score > 52), n (%)	602 (30)
Chronic pain, n (%)	763 (38.0)
Pain intensity, NRS-11^3^, mean (SD)	5.2 (2.2)
Drug treatment for pain, n (%)	368 (48.4)
Spasticity, n (%)	1025 (51.0)
Spasm intensity, NRS-11^4^, mean (SD)	3.6 (2.4)
Drug treatment for spasticity, n (%)	352 (35.8)
PDDS level, median (ICR)	2.0 (0–4)
PDDS levels (0 to 8), n (%)	
0 Normal	663 (33)
1 Mild	307 (15)
2 Moderate	280 (14)
3 Gait disabilities	243 (12)
4 Early cane	183 (9.1)
5 Late cane	94 (4.6)
6 Bilateral support	110 (5.6)
7 Wheelchair	114 (5.7)
8 Bedridden	15 (1.0)
Bowel and bladder function, n (%)	
Normal	729 (36.3)
Mild dysfunction	722 (35.9)
Moderate dysfunction	343 (17.1)
Severe dysfunction	114 (5.7)
Lost bowel or bladder function	54 (2.7)
Lost bowel and bladder function	47 (2.3)
BMI, mean (SD)	25.1 (5.1)
Smoking status, n (%)	
Daily	361 (18.0)
Occasional	117 (5.8)
Former smoker	819 (40.8)
Never smoker	712 (35.4)
Occupation, n (%)	
Employed full-time	718 (35.7)
Employed part-time	486 (24.2)
Unemployed	805 (40.1)
Personal income pr. year, n (%)	
< $30,000	467 (23.3)
$30,000 - $59,999	797 (39.7)
$60,000 - $105,000	498 (24.8)
> $105,000	123 (6.2)
Unaware	121 (6.0)
Family status, n (%)	
Married/cohabiting living without children	745 (37.1)
Married/cohabiting living with children	625 (31.2)
Living alone with children	174 (8.7)
Living alone without children	462 (23.0)
Education, n (%)	
Below high-school graduate	149 (7.4)
High-school graduate	158 (7.9)
Skilled education	414 (20.7)
Shorter courses	25 (1.2)
Short post-secondary education	235 (11.7)
Middle post-secondary education	560 (28.0)
Long post-secondary education	464 (23.1)

NRS-11, numeric rating scale (from 0 to 10); FSS, fatigue severity scale;

PDDS, patient determined disease steps; SD, standard deviation

^1^ 0 = completely dissatisfied to 10 = completely satisfied

^2^ 0 = no disturbances to 10 = high disturbed sleep

^3^ 0 = no pain to 10 = worst imaginable pain

^4^ 0 = no spasticity to 10 = worst possible spasticity

ICR, interquartile range (25–75%)

## Results

Questionnaires were returned by 2244/3606 (62%) PwMS. Of those, 235 were excluded due to incomplete questionnaires and no MS diagnosis (e.g. neuromyelitis optica), which led to an inclusion of 2009 questionnaires for analysis. The mean age was 49.4 years (range: 17 to 88 years), 69% were females, and the average time since diagnosis was 11.7 years (range: 0–66 years). More than two-thirds (69%) were women. See Table 1 for study population characteristics. Importantly, missing data were less than 1.0% for each item. For more information on data collection, study flow chart and further sociodemographic characteristics see previous paper [14].

### Prevalence of MS related symptoms and disability

Based on the PDDS score, 33% (663/2009) had mild symptoms due to MS but the symptoms did not limit activity (PDDS = 0). Sixty-two percent reported no *ambulation impairment* (PDDS = 0 to 2). Among the participants with impaired ambulation, 38% (PDDS = 3 to 8), the use of walking assistance (unilateral or bilateral support) was reported by 19% (387/2009), and 5.7% (114/2009) were wheelchair dependent. Patients who were bedridden represented 1.0% (15/2009) of the study population. Twelve percent had MS symptoms which interfered with their walking but did not need assistance to walk.

*Spasticity* was reported by 51%, with a mean intensity on NRS-11 at 2.4. The intensity of spasticity was primarily (56%) reported mild (NRS of 1–3). Among participants with spasticity, 9.0% had no spasms. The majority (77%) had mild spasms induced by stimulation or spasms occurring less than once per hour. Thirty-six percent of the participants who reported spasticity used antispasmodic drugs.

In the entire sample *bowel and bladder dysfunction* were reported by 74% and 36% reported mild dysfunction. Five percent had pronounced dysfunction including loss of bowel and/or bladder function, Table 1.

*Chronic pain* was reported by 38% with a mean intensity on NRS-11 at 5.2. Of those, 60% reported moderate pain intensity (NRS of 4–7). Five hundred and thirty-three participants with chronic pain had a DN4 score of ≥3, which is suggestable for neuropathic pain. This corresponded to 70% of those with chronic pain and 27% of the total sample. Almost half (48%) of the participants with chronic pain were using analgesics.

In total, no to mild *fatigue* (FSS < 36) was reported by 36%, moderate fatigue (FSS 36–52) by 34% and severe fatigue (FSS > 52) by 30%. No to mild *sleep disturbances* (NRS 0–3) was reported by 44%, moderate (NRS 4–7) by 41% and severe (NRS 8–10) by 15%. Only 12% (233/2009) did not experience any sleep disturbances (NRS 0).

Importantly, the prevalence of MS related symptoms and disability level did not differ when stratified by disease duration (time from MS diagnosis) in the following strata: 0–5 years; > 5–10 years; > 10–15 years, > 15–20 years; and > 20 years (data not shown).

### Correlations/associations between MS related symptoms

Table 2 shows the correlation between each MS related symptom in a Spearman’s correlation matrix. All univariate correlation analyses were statistically significant at the 0.01 level, possibly due to the large sample size; however, the correlations were primarily weak to moderate. The strength of the linear relationship was defined as follows: weak, correlation coefficients of 0 to 0.3; moderate, > 0.3 to 0.7; and strong. > 0.7 [24]. Variables with moderate positive correlation included: Spasticity and pain intensity; impaired ambulation and spasticity; fatigue and sleep disturbances; and impaired ambulation and bowel and bladder dysfunction. No variables showed strong correlation.

**Table 2:** Bivariate correlation matrix for symptom variables

Variable	Pain	Spasticity	Impaired ambulation	Sleep disturbances	Fatigue	Bowel/bladder dysfunction
Pain	1.0					
Spasticity	0.424*	1.0				
Impaired ambulation	0.300*	0.544*	1.0			
Sleep disturbances	0.292*	0.235*	0.091*	1.0		
Fatigue	0.392*	0.296*	0.291*	0.306*	1.0	
Bowel/bladder dysfunction	0.237*	0.418*	0.459*	0.150*	0.261*	1.0

*Correlation is significant at the 0.01 level (2-tailed). Bold values represent moderate strength of correlation (coefficients of > 0.3 to 0.7)

Values represent spearman rho correlation coefficients

**Fig. 1:**
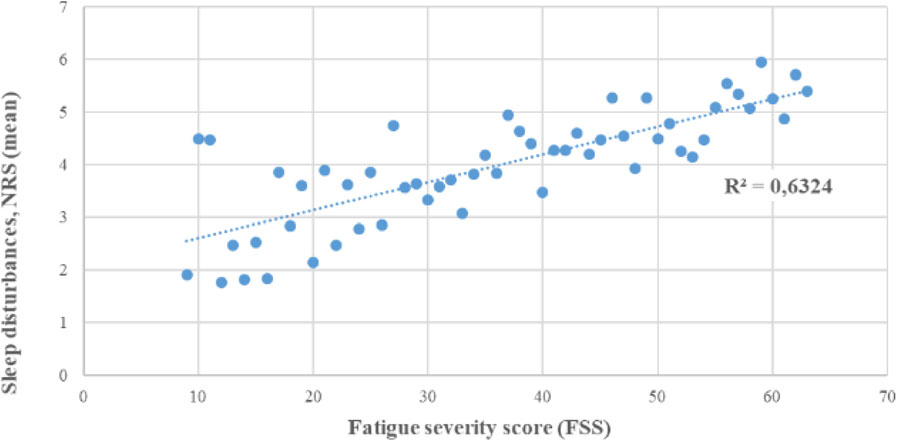
Association between fatigue severity score (FSS) and sleep disturbances. NRS, numeric rating scale: 0 = no disturbances, 10 = worst imaginable disturbances

Comparing the mean severity of sleep disturbances at each FSS score indicated a linear association, with more pronounced sleep disturbances in participants with high FSS score, Fig. 1. Calculating a linear trendline showed a R-square value of 0.63.

### Associations of MS symptoms and disability

**Fig. 2:**
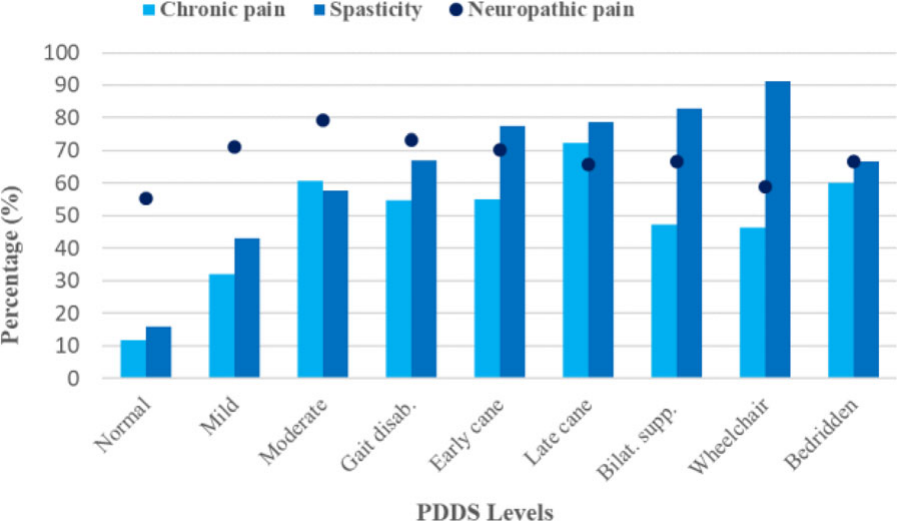
Prevalence of pain and spasticity (*n* = 2009). The dots, neuropathic pain, indicates the prevalence of neuropathic pain among responders with chronic pain

**Fig. 3:**
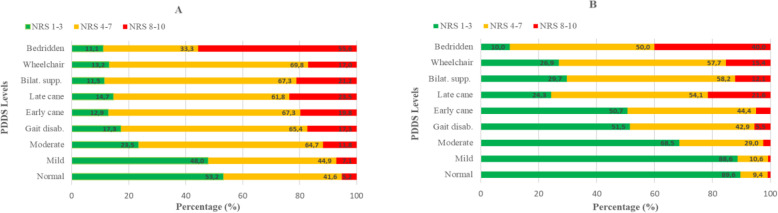
Intensity of pain (**A**) and spasticity (**B**) for each disability level. The figures include data from participants who reported chronic pain (*n* = 763) and spasticity (*n* = 1025). NRS-11, numeric rating scale. NRS-11 categorization of symptom intensity: mild (NRS 1–3); moderate (NRS 4–7); and severe (NRS 8–10). PDDS, patient determined disease steps

The prevalence of chronic pain and spasticity was less reported in participants with no to mild disability compared with participants with higher disability, Fig. 2. In the group with no disability pain was reported by 12% and spasticity by 16%. The prevalence of spasticity increased with higher disability level, as well as the intensity, Fig. 3. Participants with moderate or higher disability levels had a prevalence of chronic pain between 47 and 72%. However, the prevalence of pain did not increase to the same extent as did spasticity with higher disability. The prevalence of neuropathic pain for all disability levels ranged between 55 and 79%, Fig. 2.

**Fig. 4:**
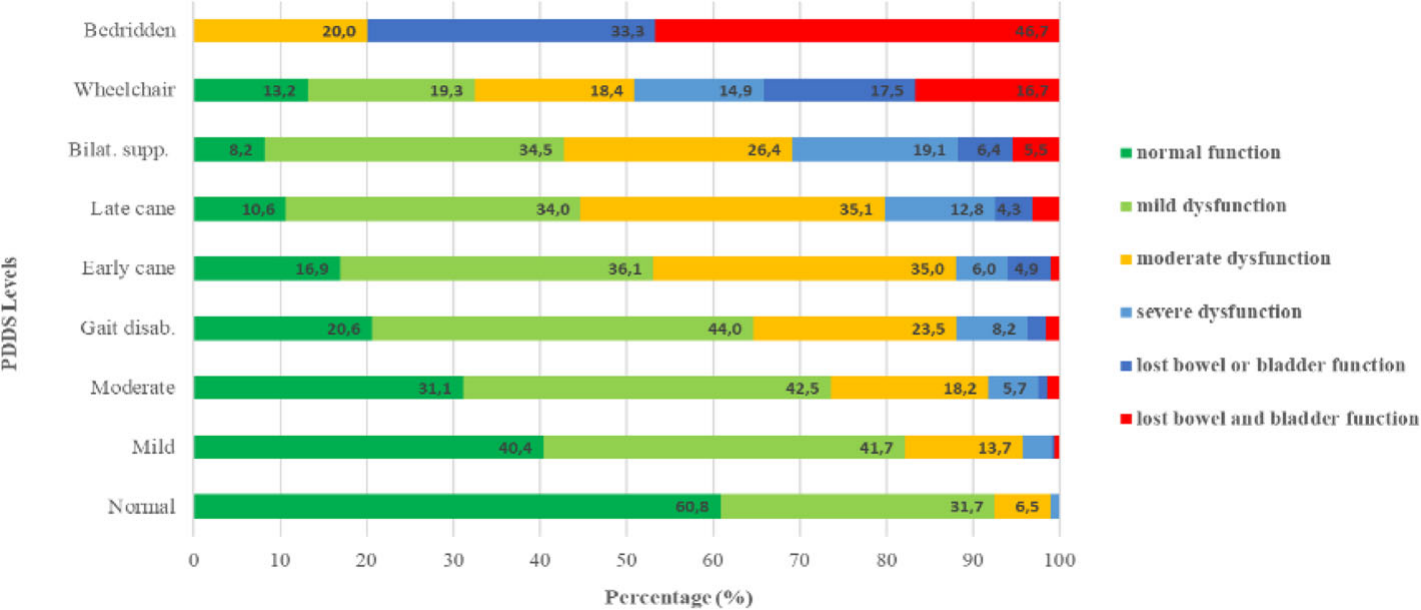
Severity of bowel and bladder dysfunction for each disability level (*n* = 2009). Category descriptions: normal; mild dysfunction (mild hesitancy, urgency and/or constipation); moderate dysfunction (moderate urinary hesitancy/. retention/urgency/ incontinence and/or must wear pads or alter lifestyle because of bowel dysfunction); severe . dysfunction (frequent urinary incontinence or intermittent self-catheterization, needs enemata or manual measures . to evacuate bowels); lost bowel or bladder function; lost bowel and bladder function. PDDS, patient determined disease steps

Bowel and bladder dysfunction became more severe with increased disability, Fig. 4. For wheelchair dependent and bedridden participants, 34 and 80% reported either loss of bowel or bladder function, or both, respectively.

**Fig. 5:**
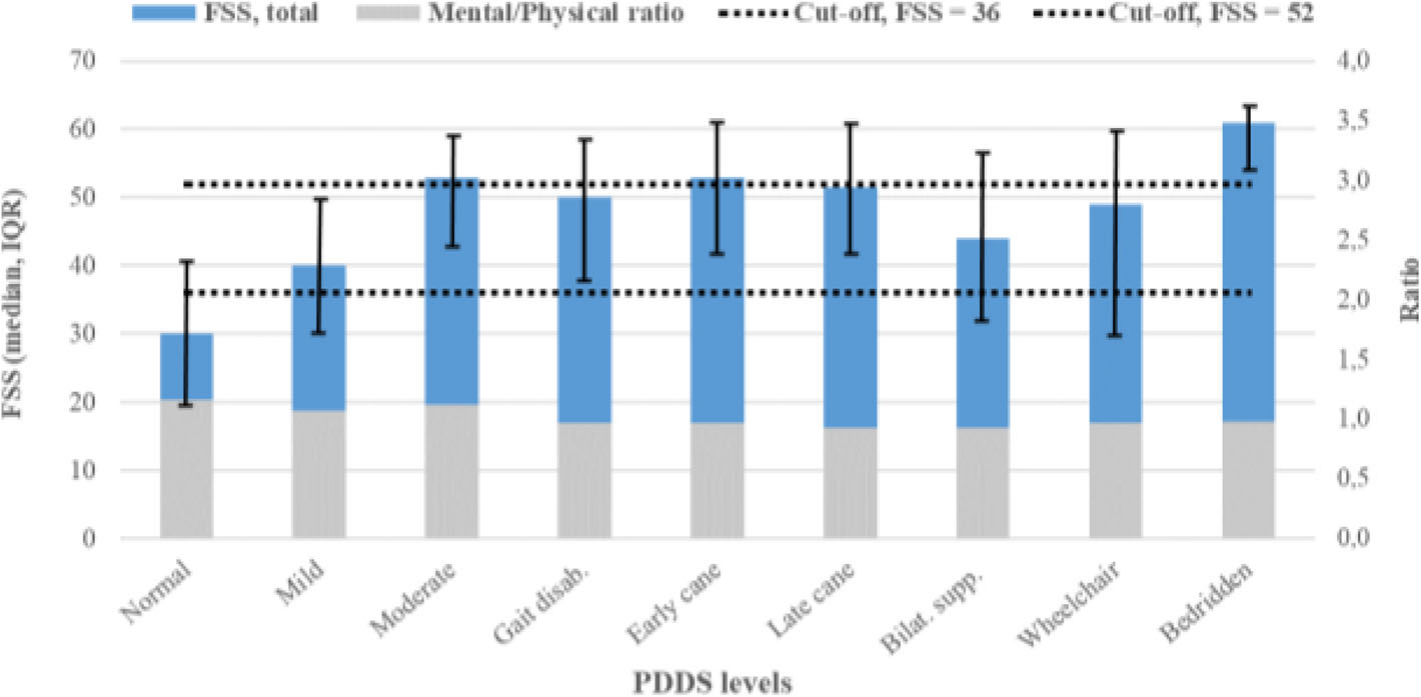
Fatigue severity score (FSS) for each disability level. The left Y axis shows the total FSS score (blue bars) and the right Y axis shows the ratio of mental and physical impact on the FSS score (grey bars). The dashed black lines represent the cut-off values of FSS: < 36: no to mild, 36–52: moderate and > 52: severe fatigue. PDDS, patient determined disease steps; IQR, interquartile range [25–75%]

Already at mild disability the FSS score was of moderate intensity, and at moderate disability the score reached severe fatigue, Fig. 5. The impact of the mental and physical components on the total FSS score was at a ratio of 0.9–1.2 throughout all disability steps, with the lowest ratio at higher disability. This indicates that the physical components had higher impact on the severity of fatigue in participants with higher disability levels.

**Fig. 6:**
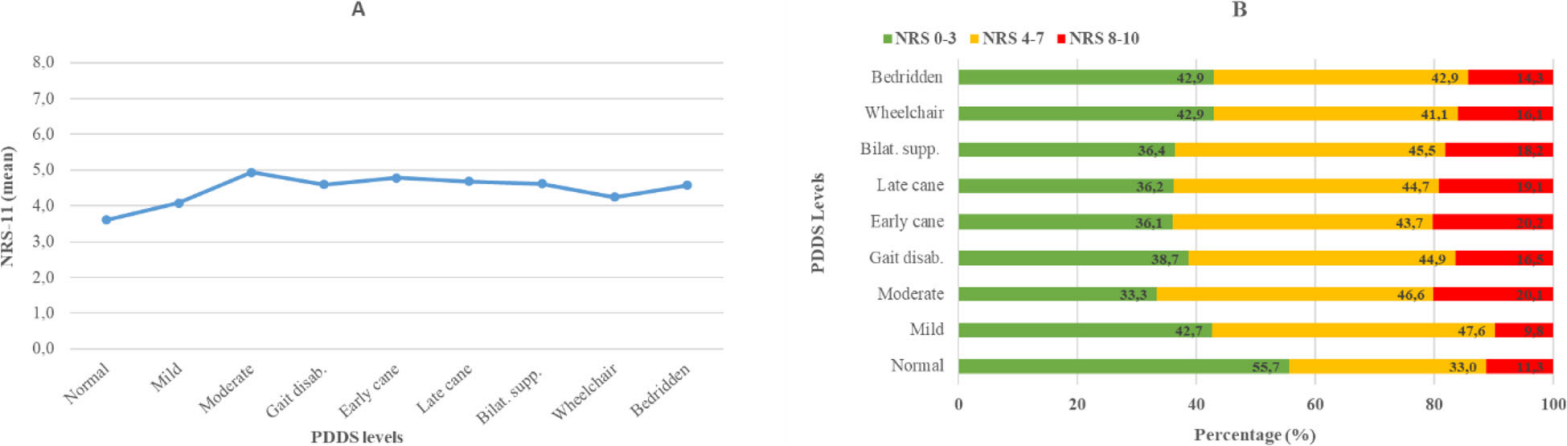
The prevalence (**A**) and intensity (**B**) of sleep disturbances at each disability level. The figures include data from all participants (*n* = 2009). NRS-11, numeric rating scale, 0 = no disturbances, 10 = worst imaginable disturbances. NRS-11 categorization: mild (NRS 0–3); moderate (NRS 4–7); and severe (NRS 8–10). PDDS, patient determined disease steps

Regarding the association between fatigue severity and disability level a similar association was found between the intensity of sleep disturbances and disability level, Fig. 6**A**. From normal to moderate disability the intensity of sleep disturbances increased; however, with higher disability the mean NRS score remained the same, and so did the intensity, Fig. 6**B**.

### Association of MS symptoms, disability and quality of life

**Fig. 7:**
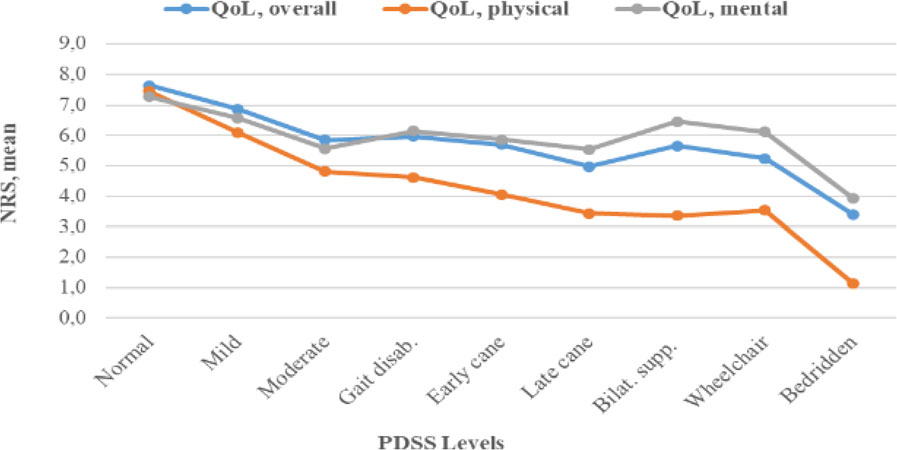
Association between quality of life (QoL) descriptors and disability level (*n* = 2009). NRS, numeric rating scale: 0 = completely dissatisfied to 10 = completely satisfied

The satisfaction on the three QoL descriptors decreased with increasing disability; however, the overall and mental satisfaction remained stable from moderate to wheelchair dependent, Fig. 7. In contrast, the satisfaction on physical health decreased almost linear with higher disability.

The multivariable linear regression model showed that all the included MS related symptoms and the disability level had statistically significant effects on the overall QoL (life situation satisfaction), Table 3. With higher symptom severity the overall QoL decreased. In addition, MS duration, occupational status and family status had significant associations. The symptoms with the strongest associations with the overall health, described by the standardized coefficient (*beta*), were fatigue (*beta* = −.232), pain (*beta* = −.145), and sleep disturbances (*beta* = −.120). For physical health: fatigue (*beta* = −.275), impaired ambulation (*beta* = −.273), and pain (*beta* = −.154) had the strongest associations. Additionally, body mass index (BMI) had statistically significant effect on physical health. Fatigue (*beta* = .239) and sleep disturbances (*beta* = .171) were the symptoms with the strongest associations with mental health. In addition, pain, bowel and bladder dysfunction, MS duration, BMI, occupational status and family status were statistically significant; however, less associated with mental health in comparison with the previous indicated MS related symptoms.

**Table 3:** Factors associated with quality of life in a multivariable regression model

	Satisfaction with life situation				Satisfaction with physical health				Satisfaction with mental health			
	B	SE	*beta*	*p*	B	SE	*beta*	*p*	B	SE	*beta*	*p*
Fatigue	−.036	.003	**−.232**	**< .001**	−.047	.004	**−.275**	**< .001**	−.039	.004	**−.239**	**< .001**
Pain	−.126	.020	**−.145**	**< .001**	−.150	.020	**−.154**	**< .001**	−.099	.022	−.106	**< .001**
Sleep disturbances	−.106	.018	**−.120**	**< .001**	−.100	.019	−.101	**< .001**	−.162	.021	**−.171**	**< .001**
Impaired ambulation	−.157	.041	−.099	**< .001**	−.482	.042	**−.273**	**< .001**	.067	.046	.040	.148
Spasticity	−.076	.026	−.074	**.003**	−.073	.026	−.064	**.006**	−.044	.029	−.041	.125
Bowel and bladder dysfunction	−.146	.049	−.068	.**003**	−.126	.050	−.053	.**012**	−.166	.055	−.073	**.002**
MS duration	.030	.006	.097	**< .001**	.013	.007	.036	.058	.035	.007	.106	**< .001**
BMI	.003	.010	.006	.774	−.037	.010	−.069	**< .001**	.024	.011	.047	**.022**
Smoking status	.028	.046	.012	.536	.035	.047	.014	.453	.035	.051	.014	.499
Occupational status	−.257	.068	−.090	**< .001**	−.107	.070	−.034	.124	−.240	.076	−.079	**.002**
Income	.013	.048	.006	.784	−.016	.048	−.006	.735	.035	.053	.014	.514
Family status	−.146	.041	−.068	**< .001**	−.031	.042	−.013	.459	−.111	.046	−.049	**.016**
Educational level	.030	.026	.024	.238	−.006	.026	−.005	.808	.054	.029	.041	.059

B, unstandardized coefficient; SE, standard error; *beta*, standardized coefficient; p, *p*-value; BMI, body mass index. Bold *p*-values represent p-values < 0.05. Underlined and bold *beta*-values represent the highest/lowest *beta*-values

## Discussion

To the best of our knowledge, this study is the largest and most comprehensive non-register-based self-report survey assessing the prevalence and severity of MS related symptoms and its association with disability and QoL. We found invisible symptoms, defined as symptoms that affect the patient greatly but are not noticed be others, including fatigue, pain and sleep disturbances highly prevalent, even at mild disability levels. Additionally, the invisible symptoms had the strongest associations with the overall QoL and were more frequent reported in our study compared with a recent large sample, registry-based study [3]. Furthermore, we addresses that some of these symptoms (i.e. sleep disturbances) may be caused by other disorders and are frequently seen in the general population, making it even more difficult to estimate the true symptom burden of MS.

In accordance with previous studies, we found that pain, fatigue and disability level were highly associated with QoL in MS [8], and that fatigue, spasticity, sleep disturbances and chronic pain are frequent symptoms in MS. [3, 4] In our study the prevalence of chronic pain and spasticity was 38 and 51%, respectively, and were primarily reported with moderate intensity of pain (NRS of 4–7) and mild intensity of spasticity (NRS 1–3).

The prevalence of pain and spasticity in our study matched the data from a German registry study [3], but the prevalence of bowel and bladder function and fatigue was higher in our study, 74% vs. 44 and 66% vs 58%, respectively. A study by Barin et al., which comprehensively addressed the symptom burden in MS among 855 MS patients from the Swiss Multiple Sclerosis Registry found slightly higher prevalences of fatigue and pain, but did not address sleep disturbances [25].

Our study population had a mean disease duration (time since diagnosis) of 11.7 years. We compared our results with those of a NARCOMS study population with a disease duration of 11–12 years [4] regarding the following symptoms (NARCOMS vs Copenhagen): Impaired ambulation (occasional use of unilateral support): 42–47% vs. 48%; fatigue (moderate) 58–60% vs. 64%; spasticity (mild) 49–53% vs. 51%; pain (mild) 48–53% vs. 38%; and bowel and bladder dysfunction (80–82% vs. 74%). It is important to notice the variation in the characterization of the symptom severity, which makes a direct comparison difficult. Therefore, possible reasons for inconsistencies across studies could be that the symptoms are registered and characterized differently, which often is an obstacle when comparing prevalences across studies. Additionally, due to the symptom variability of MS previous studies, as well as our study, have investigated selected MS symptoms. This can result in different conclusions, especially when addressing which symptom is the most frequent or when estimating the individual symptoms’ impact on or association with QoL. This clearly emphasizes the importance of including standardized patient reported outcome measures, and not only include physical disabilities, in large national and global MS registries, which also has been addressed in a study by Green el al [26]. Furthermore, other factors may influence the prevalence of symptoms observed, e.g. variations in the clinical and demographic characteristics between studies. Regarding age, sex, disease duration and disability our study population is comparable with the previous indicated studies on symptom prevalence [3, 4].

Previously, ambulation has been reported as the most valued bodily function among PwMS [27]; however, our findings, as well as a recent study [8], shows that impaired ambulation/gait difficulties primarily is highly associated with physical dimensions of QoL measures and not the overall life satisfaction. For the overall QoL we found impaired ambulation as the fourth most related factor. Possibly, MS patients with short disease duration perceive lower limb mobility as a high valued function but with longer disease duration the impact of walking ability on the overall QoL decreases, which is consistent with the association we observed between the QoL descriptors and disability level, Fig. 7. Heesen et al. reported that a greater percentage of MS patients with a disease duration of < 5 years had ranked walking as the most valued function compared to MS patients with a disease duration of > 15 years; although walking was in both groups the highest ranked function.

With the exception of fatigue and sleep disturbances, all the included MS related symptoms increased in severity with higher disability level. Fatigue was reported moderate to severe already at moderate disability, which is consistent with a previous study [11], and furthermore highlight the significant importance of fatigue in MS.

The reported prevalence of chronic pain was found higher in our study in comparison with the general Danish population [28]. In a review article from 2012 by Harker et al. the estimated prevalence of noncancer pain in the general population in Denmark was 20, and 16% had moderate to severe chronic pain, similar to other European countries. In 2017 The Danish Authority conducted a national health survey (Danes’ Health - The National Health Profile) among > 300.000 Danish citizens aged 16 or more [29]. The overall prevalence of self-reported chronic pain was 29%. Additionally, 19 and 14% reported severe fatigue and sleep disturbances, respectively. We found a prevalence of severe fatigue and sleep disturbances at 30 and 15%, respectively, which indicates that sleep disturbances also are common among the general population and possibly caused by other conditions than MS.

The relatively large population size (*n* = 2009), the relatively high response rate (62%) and the low occurrence of missing data (< 1% for each variable) are major strengths of this study. In addition, our study population was comparable with regard to age, sex and disability to the DMSC population registered in the Danish Multiple Sclerosis Registry. This reduces the likelihood of selection bias, an often inevitable bias in questionnaire surveys (please see original paper for further details) [14]. Furthermore, we included sociodemographic characteristics, which in previous studies have shown association with QoL. Most of the questions covered the preceding 3 months, why potential recall bias is limited. Our study provides clearly defined characterization and descriptions of the MS related symptoms, which often is a limitation in registry studies. Registry studies are often a repository of data derived from clinicians, which is why data on patients with no regular contact to their physician are limited and may generate selection bias. In addition, some MS registries are solely based on volunteer patient reported outcomes; however, the combination of clinician and patient reported outcomes are increasingly being included in MS registries.

A major limitation was we were not able to obtain information on cognition and mental disorders, which have been found to be a predictor for QoL in MS. [8] Therefore, we were not able to cover the whole symptomatology of MS; however, we were able to comprehensively address the prevalence of multiple selected symptoms and their associations with disability and QoL.

In addition, the instrument used to measure QoL (QoL-BDS), including the translated Danish version, has not been validated in MS; however, QoL-BDS has been widely used among patients with spinal cord injury and has recently been psychometrically tested, providing evidence of reproducibility [30]. The instrument was initially included in the questionnaire to strengthen the comparability with previous data from the questionnaire used among patients with spinal cord injury in Denmark [31, 32]. The instrument was therefore prior to our study translated into Danish. In this study, the authors found the instrument applicable to MS based on the assumption that the selected symptoms (such as chronic pain, spasticity, sleep disturbances) have similar impact on QoL in PwMS and people with spinal cord injuries. Surveys conducted electronically can limit the availability for people with inadequate technical skills, or severe physical or cognitive impairment. Despite this limitation our study did include participants with high disability levels. Additionally, due to the cross-sectional study design we cannot rule out the inevitable limitation of reverse causation bias when assessing the selected variables’ association with QoL.

In conclusion, we found invisible symptoms highly prevalent, even at mild disability levels. Fatigue, sleep disturbances and pain were highly associated with the overall QoL and were more frequently reported in our study compared with previous registry-based studies. These symptoms may be underreported in registries based on clinician reported outcomes, possibly because some of these symptoms, e.g. sleep disturbances, are frequent in the general population and may be caused by other conditions than MS. This emphasizes the importance of including standardized patient reported outcomes in nationwide registries to better understand the impact of the symptom burden in MS.

### Availability of the questionnaire

The questionnaire used in the current study was developed to assess the cannabis use among PwMS in Denmark but also comprehensively included information on selected MS related symptoms, clinical and sociodemographic characteristics. Data on cannabis use have previously been published [14] but the questionnaire has not been published in its entirety. The questionnaire is available from the corresponding author on reasonable request.

## Data Availability

The datasets generated and analysed during the current study are available from the corresponding author on reasonable request.

## Abbreviations

DMSC: Danish Multiple Sclerosis Center
DN4: Douleur Neuropathique 4
EDSS: Expanded disability status scale
FSS: Fatigue severity scale
MS: Multiple sclerosis
NARCOMS: North American Research Committee on Multiple Sclerosis
NRS: Numeric rating scale
PDDS: Patient determined disease steps
PPMS: Primary progressive multiple sclerosis
PwMS: People with MS
QoL: Quality of life
QoL-BDS: Quality of life basic data set
RRMS: relapsing-remitting multiple sclerosis
SPMS: Secondary progressive multiple sclerosis
